# Outcomes of endoscopic and microscopic transsphenoidal surgery on non‐functioning pituitary adenomas: a systematic review and meta‐analysis

**DOI:** 10.1111/jcmm.13445

**Published:** 2018-01-04

**Authors:** Shi‐Yuan Yu, Qiu Du, Si‐Yuan Yao, Ke‐Nan Zhang, Jian Wang, Zhe Zhu, Xiao‐Bing Jiang

**Affiliations:** ^1^ State Key Laboratory of Oncology in South China Collaborative Innovation Center for Cancer Medicine Department of Neurosurgery Sun Yat‐sen University Cancer Center Guangzhou China; ^2^ Zhongshan School of Medicine Sun Yat‐sen University Guangzhou China; ^3^ Department of Histology and Embryology Zhongshan School of Medicine Sun Yat‐sen University Guangzhou China; ^4^ Division of Regenerative Medicine Department of Medicine University of California San Diego School of Medicine La Jolla CA USA; ^5^ Department of Stem Cell Biology and Regenerative Medicine Lerner Research Institute Cleveland Clinic Cleveland OH USA

**Keywords:** meta‐analysis, nonfunctioning pituitary adenoma, endoscopic, microscopic, transsphenoidal

## Abstract

Both microscopic and endoscopic transsphenoidal surgery are effective approaches for nonfunctioning pituitary adenomas. The issue on the comparison of their efficacy and safety remains inconsistent. A thorough search of the literatures (PubMed, EMBASE, MEDLINE) were performed up to March 2017. Studies reporting outcomes of microscopic or endoscopic transsphenoidal surgery on nonfunctioning pituitary adenomas were included. A meta‐analysis was performed focusing on the early stage and long term outcomes. The final search yielded 19 eligible studies enrolling 3847 patients, 389 of them underwent microscopic approach and 3458 of them with endoscopic approach. As to the early stage outcomes, the rate of gross tumor resection was significantly higher in the endoscopic group than that in microscopic group (73% *versus* 60%, *P* < 0.001). Meanwhile, endoscopic approach showed priority over microscopy on postoperative hypopituitarism (63% *versus* 65%, *P* < 0.001) and CSF leakage (3% *versus* 7%, *P* < 0.001). For the long term outcomes, the rate of visual improvement was significant higher in the endoscopic group than that in microscopic group (77% *versus* 50%, *P* < 0.001). However, there was no significant difference between the groups regarding the rate of permanent diabetic insipidus and meningitis. The endoscopic approach may be associated with higher rate of gross tumor movement and lower risk of postoperatively complications for treating nonfunctioning pituitary adenoma, when compared with microscopic approach. However, the confidence was shorted due to limited high quality evidence (largely randomized and controlled studies).

## Introduction

Non‐functioning pituitary adenoma (NFPA) is the most common phenotype of pituitary adenomas with considerable morbidity because of hypopituitarism and mass effect 1. In contrast to the prolactin and growth hormone‐secreting pituitary adenomas, none of effective drugs are available for NFPA, and transsphenoidal surgical resection remains the first‐line treatment 1. However, total resection of these lesions is challengeable because of the involvement of cavernous sinus. Moreover, the tumour‐associated hypopituitarism is another serious issue needed to be addressed, as the post‐operative hypopituitarism has been shown to contribute to an overall excess mortality in women 2. Therefore, surgical resection is significantly vital for the prognosis of patients with NFPA, to increase the rate of gross total resection (GTR) and eliminate the frequency of post‐operative hypopituitarism are the two most important issues needed to be addressed 1.

The microscopic transsphenoidal approach has long been taken as a standard procedure to manage NFPA, which has been challenged by the popular of endoscopic approach 3. With improved visualization, endoscopic approach provides a wider and superior picture of the parasellar and suprasellar compartments, which may gain access to improve the rate of GTR, protection of normal pituitary tissue, and thus reduce post‐operative complications 3. Previous studies have systemically reviewed the outcome of endoscopic and microscopic approach among either all the subtypes of pituitary adenomas 4, 5 or growth hormone‐secreting pituitary adenomas 6. As we mentioned before, NFPA is a unique entity, where cavernous compartments are more commonly involved and surgical resection plays a much more crucial role. However, whether endoscopic approach demonstrates priority over microscopic approach in treating NFPA remains inconsistent 7. We thus performed a meta‐analysis focusing on the outcome and complications of microscopic and endoscopic transsphenoidal resection of NFPA.

## Materials and methods

### Literature search strategy

A systematic search of PubMed, EMBASE, MEDLINE and references of all the retained studies were reviewed for any related articles ranging from the January 2000 to March 2017. The following searching terms were used to extract all the relative articles, non‐functioning, pituitary, neoplasm, adenoma, endoscopic, endoscopy, microscopic, transsphenoidal and surgery.

### Inclusion and exclusion criteria

Studies were included if they matched the following criteria, (*i*) A comparison article between endoscopic and microscopic approach for non‐functioning pituitary adenoma. (*ii*) Retrospective studies or single‐armed studies observing the outcome of either endoscopic or microscopic approach. (*iii*) Studies consisted of no less than 10 samples. (*iv*) Duplicated reports of the same population or increased lengths of follow‐up, only the most complete studies were included. Studies were excluded if they had any of the following characteristics, (*i*) Endoscopic‐assisted comparison studies. (*ii*) Studies lack information of the efficacy and complications following operation. (*iii*) Studies published in other languages except English.

### Outcome interests and statistics analysis

The early stage (GTR, CSF leak, hypopituitarism and meningitis) and long‐term outcomes (visual improvement and permanent diabetic insipidus (DI)) after the resection of NFPA were systemic evaluated.

Chi‐square test and *I* ^2^ were used to evaluate the heterogeneity among the studies towards each index. A random effect model or fixed effect model was applied for meta‐analysis with (*P* < 0.05, *I* ^2^ > 50%) or without heterogeneity (*P* > 0.05, *I* ^2^ < 50%), respectively. The comparison of the outcome between the endoscopic and microscopic approaches was tested using SPSS 19.0 (Chicago, IL, USA), *P* < 0.05 was considered as significant difference. Systematic analysis was conducted with the software, Review Manager 4.2 (Revman, The Cochrane Collaboration, Oxford, UK).

## Results

A total of 824 articles were initially included, 745 of them were removed as a result of unrelated content or not published in English. Then, the left 79 articles went through full‐text reviewing. Eventually, 19 studies were included in the final analysis, enrolling 389 patients underwent microscopic transsphenoidal resection and 3458 patients with endoscopic approach. Four of which directly compared endoscopic and microscopic approaches 7, 8, 9, 10, while the other 15 articles are single‐armed studies 11, 12, 13, 14, 15, 16, 17, 18, 19, 20, 21, 22, 23, reporting purely endoscopic or microscopic transsphenoidal surgery on NFPA 11, 12, 13, 14, 15, 16, 17, 18, 20, 21, 22, 23. The proportion of gender, the presence of pre‐operatively hypopituitarism, visual deficiency and invasive tumours were significant different between the two groups (Table 1).

**Table 1:** Patient and tumour features in studies on endoscopic/microscopic approach for non‐functioning pituitary adenomas

	Number of endoscopic/microscopic studies	Number of endoscopic/microscopic patients	Endoscopic pooled proportion (95%CI)	*I* ^2%^ (*P* value for heterogeneity)	Microscopic pooled proportion (95%CI)	*I* ^2%^ (*P* value for heterogeneity)	*P* value for difference
Patient characteristics							
Age	11/5	1381/389	57.73 (51.35–56.10)	0 (0.972)	56.22 (54.07–58.37)	0 (0.971)	0.580
Males%	19/5	3458/389	57 (54–60)	62.8 (<0.001)	63 (58–68)	17.1 (0.305)	0.024
Presenting symptoms							
Hypopituitarism%	8/3	892/269	48 (28–68)	97.9 (<0.001)	23 (9–37)	86.5 (0.001)	<0.001
Pre‐operative visual deficit%	13/2	2200/226	57 (49–65)	92.7 (<0.001)	67.4 (0.080)	44 (33–56)	<0.001
Tumour characteristics							
Macroadenoma%	7/2	1030/167	86 (76–95)	77.5 (0.012)	63 (53–73)	0 (<0.001)	<0.001
Superasellae invasion%	4/1	967/144	53 (6–100)	99.5 (<0.001)	11	NA	<0.001
Intrasellae/Clinvus invasion%	3/1	476/144	55 (−20–131)	99.5 (<0.001)	12	NA	<0.001
Knosp score 0–2%	10/4	2216/354	45 (36–55)	94.9 (<0.001)	45 (29–60)	89.4 (<0.001)	0.979
Knosp score 3–4%	10/4	2216/354	55 (45–64)	94.9 (<0.001)	55 (40–71)	89.4 (<0.001)	0.979
Maximum tumour diameter (mm)	9/2	1576/226	27.15 (16.35–37.95)	0 (0.971)	27.65 (10.50–44.80)	0 (0.868)	0.548

**Figure 1:**
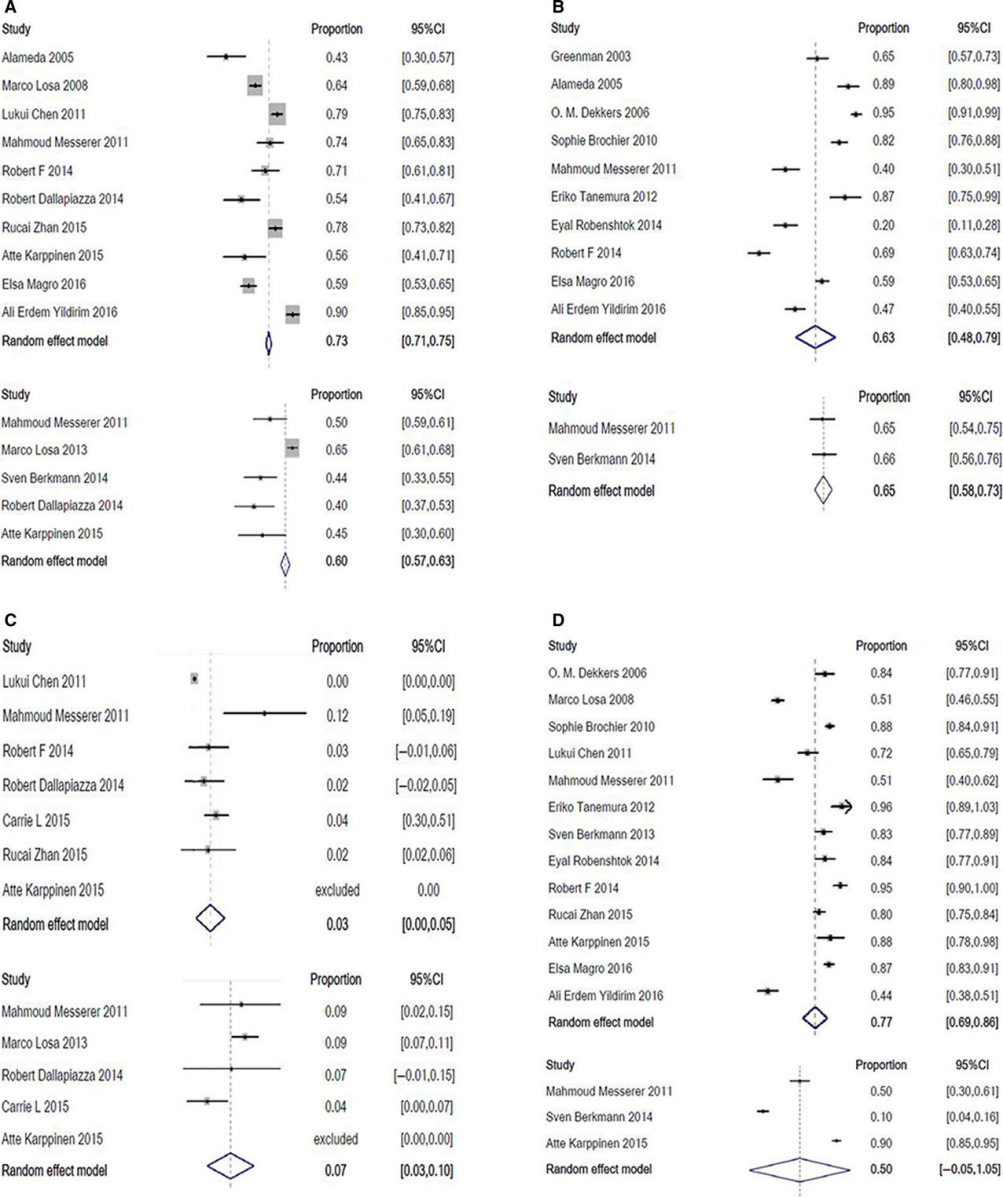
Pooled proportion of GTR (**A**), hypopituitarism (**B**), CSF leak (**C**) and postoperative visual improvement (**D**) of endoscopic (upper) and microscopic (lower) transsphenoidal resection of non‐functioning pituitary adenomas.

For the earl‐stage outcome, the rate of GTR was significantly higher in the endoscopic group than that in the microscopic group (*P* < 0.001; Fig. 1A). The rate of hypopituitarism (Fig. 1B) and CSF leak (Fig. 1C) in endoscopic group were less likely present than that in the microscopic group (*P* < 0.001). As to the long‐term outcome, patients endoscopically treated had a significantly higher rate of visual improvement than those treated with microscopy (*P* < 0.001; Fig. 1D). The rate of post‐operative permanent DI was comparable in the two groups.

## Discussion

The merits and disadvantages of endoscopic and microscopic transsphenoidal pituitary adenoma resection have been evaluated in several previous studies 4, 5, 6. However, the comparison of endoscopic and microscopic transsphenoidal resection specifically on NFPA has never been systematically performed before. In this study, the pooled data showed that patients with NFPA endoscopically treated tended to have higher rate of GTR and visual improvement, as well as lower percentage of post‐operative hypopituitarism and CSF leak.

Similarly, in the meta‐analysis performed by Gao *et al*., the rate of GTR was higher in the endoscopic group than that in the microscopic group, as well as less frequency perforation in patients underwent endoscopic surgery 4. In consistence, Li *et al*. concluded that endoscopic transsphenoidal surgery was associated with higher incidence of GTR 5. A major prognostic factor for the resection outcome and recurrence of a pituitary adenoma is its parasellar extension, particularly into the space of the cavernous sinus. With the panoramic visualization, endoscopy provides a wider and superior pathway to the area of parasellar and suprasellar compartments, which may gain access to improve the rate of GTR, protection of normal pituitary tissue and thus reduce postoperative complications 5.

Benefit from the progress of reconstructive techniques, the incidence of post‐operative CSF leak obviously decreased 24. In addition, endoscopy appeared to be more sensitive to identify CSF leak intra‐operatively and perform reconstruction of sellae 6. This may help to explain the lower rate of postoperatively CSF leak in endoscopic group in this study.

Still, we should note that our systematic review and meta‐analysis still have some limitations in need of further investigation. First, significant heterogeneity was noted in the majority of the studies in baseline information of patients (Table 1). Second, a large number of related studies were excluded from this studies, as they enrolled all subtypes of pituitary adenomas, where the particular data on NFPA were not available. Apart from this, the significant weakness of this study was lack of evidence of grade II and III. Thus, more randomized trials with large‐scale cohort of patients are warranted to help determine the efficacy of microscopic and endoscopic transsphenoidal surgery in treating NFPA.

## Conflict of interest

The authors declare that they have no conflict of interest.
